# Factors associated with generalised anxiety disorder and depression among adults living with diabetes and hypertension comorbidity in rural Bangladesh: findings from a cross-sectional study

**DOI:** 10.1136/bmjopen-2025-102000

**Published:** 2025-09-05

**Authors:** Meghna Chakravartty, Md. Mashuk Shahriar Shuvo, Sita Kumari, Tanni Chakma Jhilik, Tanmoy Sarker, Fahmida Akter, Md Mokbul Hossain, Ali Ahsan, Mahbub Latif, Malay Kanti Mridha

**Affiliations:** 1 Centre for Non-communicable Diseases and Nutrition, BRAC University James P Grant School of Public Health, Dhaka, Bangladesh; 2 Statistical Research and Training, Dhaka University, Dhaka, Bangladesh

**Keywords:** Hypertension, Anxiety disorders, Depression & mood disorders, Multimorbidity, General diabetes

## Abstract

**Abstract:**

**Objective:**

The rising burden of non-communicable diseases (NCDs), including mental health disorders (MHDs) such as anxiety and depression, poses a significant public health challenge globally. Evidence suggests that both diabetes and hypertension, the two most prevalent NCDs, are linked to a higher prevalence of MHDs. However, there is a lack of evidence on prevalence of generalised anxiety disorder (GAD) and depression among adults living with both diabetes and hypertension in Bangladesh. We aimed to assess the prevalence of GAD and depression and explore the associated factors among adults living with diabetes and hypertension comorbidity in rural Bangladesh.

**Design:**

We implemented a cross-sectional study.

**Setting:**

The study was conducted in Chirirbandar, a sub-district of Dinajpur, Bangladesh.

**Participants:**

We interviewed a total of 387 adults living with diabetes and hypertension comorbidity.

**Primary outcome measures:**

We had two primary outcome measures: GAD and depression. Individuals scoring ≥10 on the General Anxiety Disorder-7 scale were considered as having GAD and individuals scoring ≥10 on the Patient Health Questionnaire-9 scale were considered as having depression. The outcome variables were dichotomised based on these scores.

**Results:**

The prevalence of GAD was 7.24% (95% CI 5.04 to 10.29). Education level (grades 5–9) (adjusted OR (AOR): 3.40, 95% CI 1.26 to 9.19) and household wealth status (highest wealth tertile) (AOR: 0.12, 95% CI 0.02 to 0.62) were associated with GAD. The prevalence of depression was 17.83% (95% CI 14.32 to 21.98). Socioeconomic factors associated with depression included unemployment (AOR: 3.26, 95% CI 1.05 to 10.10) and household wealth status (highest wealth tertile) (AOR: 0.45, 95% CI 0.21 to 0.98). Higher odds of depression were also observed among participants with controlled hypertension (AOR: 3.88, 95% CI 1.81 to 8.35). Other factors, such as tobacco use, dietary diversity and physical activity, were not associated with GAD or depression.

**Conclusion:**

A high prevalence of GAD and depression was observed among adults living with diabetes and hypertension comorbidity. The findings from the study emphasise the need for integration of mental health services into the existing non-communicable disease care. The identified factors associated with GAD or depression should be considered to develop targeted interventions for people with hypertension and diabetes comorbidity in Bangladesh.

**STRENGTHS AND LIMITATIONS OF THIS STUDY**

- Generalised anxiety disorder (GAD) and depression were measured by using valid and reliable tools: General Anxiety Disorder-7 and Patient Health Questionnaire scales, respectively.
- As it was a cross-sectional study, we could not establish any causal relationship between the factors and GAD or depression.
- Data on lifestyle factors and mental health were collected based on self-reports, and therefore, the possibility of information bias cannot be ruled out.

## Introduction

 Non-communicable diseases (NCDs), such as cardiovascular diseases, cancer, diabetes and chronic obstructive pulmonary disease along with mental health disorders (MHDs), are responsible for nearly three-quarters of deaths in the world.^1^ The rising prevalence of NCDs is a growing concern globally, and addressing the burden of NCDs is relevant for achieving the Sustainable Development Goals. Diabetes and hypertension are among the most common NCDs in low- and middle-income countries (LMICs) that often share overlapping risk factors.^2 3^ The International Diabetes Federation estimated that in Bangladesh, 13.1% of adults had diabetes in 2021, and the prevalence is expected to be almost double by 2045.^4^ On the other hand, nearly 20% of adults were affected by hypertension in Bangladesh in 2021.^5^ Moreover, there is evidence of higher prevalence of hypertension among the known diabetic patients.^6^ The prevalence of diabetes-hypertension comorbidity is 4.5% among Bangladeshi adults.^7^ This coexistence of diabetes and hypertension can lead to serious health outcomes like heart attacks and stroke, kidney function impairment, etc. Adequate management and control over both conditions are neces thesary for prevention of those health complications.^8^

On top of the rising burden of NCDs, MHDs are a growing concern. In LMICs, the burden of disease due to MHDs, such as anxiety and depression, along with other NCDs is high.^9^ According to WHO, 23% of people with anxiety disorders and 27% of all people with depressive disorders are residents of Southeast Asia.^10^ According to the National Mental Health Survey 2019, the prevalence of MHDs in Bangladesh was 18.7% among adults, making it a major public health issue.^11^ The most prevalent MHDs were depression (6.7%) and anxiety disorders (4.7%).^11^ The Ministry of Health and Family Welfare of Bangladesh included MHDs under Non-Communicable Disease Control programme.^11^

Both diabetes and hypertension are linked to a higher prevalence of MHDs.^312^,^14^ A systematic review and meta-analysis conducted in 2023 estimated that the pooled prevalence of depression was 42% among diabetic patients in Bangladesh.^15^ Moreover, recent longitudinal and cross-sectional studies conducted across various regions show increasing evidence of a positive association between hypertension and anxiety.^16^ While studies have explored the prevalence of MHDs among individuals with NCDs globally, few address the prevalence of GAD and depression among population living with diabetes and hypertension comorbidity. Moreover, studies that estimated MHDs among adults living with comorbidities are mainly from the USA, Europe and Australasia,^9^ underscoring the importance of research in LMICs, including Bangladesh. Additionally, there is limited information on demographic, socioeconomic and lifestyle factors that might influence these MHDs among individuals with comorbidities living in rural Bangladesh. Accordingly, in this study, we aimed to assess the prevalence of GAD and depression among people living with diabetes and hypertension comorbidity along with an exploration of the associated demographic, socioeconomic and lifestyle factors.

## Materials and methods

### Study design and study setting

This cross-sectional study was conducted from 28 October 2024 to 5 January 2025, in Chirirbandar sub-district, Dinajpur, Bangladesh. There is an ongoing project titled ‘Understanding the Patterns and Determinants of Health in South Asian People: South Asia Biobank’ by BRAC James P Grant School of Public Health (BRAC JPGSPH) as a part of strengthening NCDs surveillance system in Bangladesh. Considering the availability of a sampling frame of adults living with diabetes and hypertension comorbidity, Chirirbandar sub-district was chosen for our study.

### Participants

The participants of our study were individuals aged 18 years or above**,** diagnosed with both diabetes and hypertension and living in the study area for at least 12 months. Individuals with severe physical disabilities, unable to speak or having cognitive impairments that prevent effective participation in the study were excluded from the study. Additionally, currently pregnant women were excluded.

### Sampling

Assuming a prevalence rate of 50% (GAD and depression), using a 95% CI and 5% margin of error, we estimated the sample size n=384, using $n=\frac{Z^{2}p\left(1-p\right)}{d^{2}}$formula, where *Z* represents the z-score corresponding to 95% level of confidence, *p* is the prevalence and *d* is the margin of error. After adjusting for a 20% non-response rate, the final sample size was 480.

As a part of the ‘South Asia Biobank’ study, the ongoing NCD surveillance system of BRAC JPGSPH in Chirirbandar had identified population with diabetes or hypertension or diabetes and hypertension comorbidity through blood pressure and fasting blood glucose measurements, following WHO criteria.^17^ From this pre-identified population, we created a sampling frame comprising 481 people who had diabetes and hypertension comorbidity. As our final sample size was 480, we approached all 481 people from the sampling frame and recruited participants according to previously described exclusion and inclusion criteria. Among the 481 eligible participants in the sampling frame, 30 were dead, 46 were out of the study area during the data collection period, 12 refused to participate and 6 individuals were excluded due to their physical disabilities or cognitive impairment. Accordingly, we collected data from 387 individuals and all those were included in the final analysis.

### Outcome measures

Our outcome variables were GAD and depression. A structured pre-tested questionnaire in Bangla was used, incorporating General Anxiety Disorder-7 (GAD-7) scale for anxiety assessment and Patient Health Questionnaire-9 (PHQ-9) for depression assessment. The GAD-7 scale is a 7-item self-report questionnaire, each item rated on a 4-point Likert scale ranging from 0 (‘not at all’) to 3 (‘nearly every day’), with a total score range of 0–21. Individuals scoring ≥10 on the GAD-7 scale were considered to have GAD.^18^

The PHQ-9 is a 9-item scale designed to assess depressive symptoms, also rated on a 4-point Likert scale (0–3), resulting in a total score between 0 and 27. Individuals scoring ≥10 on the PHQ-9 scale were considered to have depression.^19^ Both outcome variables were dichotomised based on these scores.

PHQ-9 and GAD-7 both have been widely used globally and in South Asian settings.^9 12 13 20^ In Bangladesh, the Bangla version of both tools has previously been applied in population-based studies and demonstrated acceptable psychometric properties.^21 22^ GAD was assessed as a distinct anxiety disorder using the GAD-7 because it is a well-established, specific screening tool. PHQ-9 captures a broader spectrum of depressive symptoms rather than diagnosing specific subtypes of depression such as major depressive disorder or persistent depressive disorder. In alignment with similar epidemiological studies, our aim was to capture individuals with generalised anxiety or depressive symptoms who might benefit from mental health interventions, rather than to establish formal clinical diagnoses. Therefore, in our study, depression was measured more broadly using PHQ-9 without classifying specific subtypes, while GAD was assessed using the GAD-7.

### Other measures

Through reviewing the relevant literatures, we also administered data collection tools for capturing sociodemographic information, behavioural risk factors, self-care practices, medical history of diabetes and hypertension, and clinical variables, including body mass index (BMI), blood pressure and blood sugar level (online supplemental table 1). Sociodemographic information included respondent’s age, sex, education level, marital status and employment status. For generating household wealth status, data on materials of floors and walls of the houses, supply of electricity and asset ownership were considered. Using the tools of WHO STEPwise approach to NCD risk factor surveillance, we also collected data on tobacco consumption, fruits and vegetable intake, physical activity, alcohol consumption and indoor air pollution. Additional data were collected on dietary habit using the Minimum Dietary Diversity for Women questionnaire. For details of the categories/measurements of explanatory variables of this study, please see online supplemental table 1.

### Data collection

The data collection team comprised four research team members, three trained research assistants and one data collection supervisor. As the research assistants were previously trained on similar research tools, a 2-day-long in-person training was imparted to train and standardise the research assistants and research team members. Interviews were conducted and measurements of weight, blood pressure and random blood glucose level were performed following standard procedure and maintaining privacy of the participants. We informed the government authorities beforehand about our study for their support and cooperation, if needed. We called the participants using their phone number and invited them to a designated location (community clinic or school settings) for the interview and measurements. Participants, who could not visit the designated location, were approached at their households. Written informed consent was taken from the participants prior to the interviews assuring the confidentiality and anonymity. Data collection was done using electronic devices (mobile phones/tablet computers) employing forms developed in Kobo Toolbox application to ensure real-time data entry.

Blood pressure was measured in the right arm keeping participants at a resting state for at least 30 min and using digital blood pressure machines (Omron HEM-7120). During the blood pressure measurements, we also ensured that participant’s urinary bladder was empty and he/she did not consume any kind of tobacco products or betel leaves, any tea or coffee for at least 30 min prior to the measurement. After ensuring proper sitting position, two measurements were recorded at 1 min interval and if the difference between the two measurements was >10 mm Hg, a third measurement was taken. Random blood glucose level was assessed using a glucometer (ACCU-CHEK Instant) following all aseptic precautions. To measure participants’ weight, two measurements were taken by using a digital weighing scale (TANITA UM-070). If the difference between the first two measurements exceeded 0.1 kg, a third measurement was taken. The closest two values were determined for the mean value. Participants were instructed to remove heavy clothing or accessories before weight measurement. The height of the participants was pooled from the NCD surveillance study.

To ensure accuracy, all instruments were calibrated each morning before the data collection. Each day, after the data collection, team meetings were held to review the process. Responses and measurements were cross-checked by the research team members and research assistants to ensure data accuracy and reliability.

### Statistical analysis

We conducted data analysis using software STATA V.17.0 (StataCorp, TX, 77845, USA). We used descriptive analysis to summarise the characteristics of study population. Univariate analysis was done to measure the prevalence of GAD and depression. To evaluate the association between outcome variables and the explanatory variables, logistic regression was used. The variables, which showed association with GAD and depression with p value ≤0.2 in the unadjusted models, were selected for multivariable logistic regression analysis.^21 23^

### Patient and public involvement

Patients or the public were not involved in the design, conduct, reporting or dissemination plans of our research.

## Results

Among 481 participants approached, 387 completed the interview, resulting in a response rate of 80.46%. The mean age of the participants was 56.99 years (± 0.56) with 42.12% of our participants from the older adult (60 years and above) age group, and 28.68% from the 50–59 years old age group. We had more female participants (61.76%) than male participants (38.24%). Most of the participants were currently married (85.79%). The majority of the participants were homemakers (53.23%), 32.30% were currently employed and 6.72% were unemployed for at least the last 1 year, and the rest (7.75%) were retired. In terms of educational background, 30.49% had secondary or above level education (grade ≥10) and 42.89% had no education or incomplete primary education (grades 0–4) (table 1).

**Table 1:** Background characteristics of the study participants (n=387)

Characteristics	Categories	Frequency (n)	Percentage (%)
Sociodemographic			
Age (years)	**Mean (± SD) =** 56.99 (± 0.56)		
	18–39	30	7.75
	40–49	83	21.45
	50–59	111	28.68
	60 and above	163	42.12
Sex	Male	148	38.24
	Female	239	61.76
Marital status	Currently married	332	85.79
	Others	55	14.21
Employment	Currently employed	125	32.30
	Homemaker	206	53.23
	Currently unemployed	26	6.72
	Retired	30	7.75
Religion	Islam	344	88.89
	Others	43	11.11
Education level	Grades 0–4	166	42.89
	Grades 5–9	103	26.61
	Grade ≥10	118	30.49
Household wealth status	Lowest	130	33.59
	Middle	128	33.07
	Highest	129	33.33
Behavioural risk factors			
Tobacco (smokeless/smoking) consumption	No	292	75.45
	Yes	95	24.55
Minimum dietary diversity	Inadequate	199	51.42
	Adequate	188	48.58
Processed food intake	Consumption of savoury snacks		
	No	263	67.96
	Yes	124	32.04
	Consumption of sweet		
	No	275	71.06
	Yes	112	28.94
	Consumption of sugar sweetened beverages		
	No	278	71.83
	Yes	109	28.17
Excess salt intake	Always/often	53	13.70
	Sometimes to rarely	145	37.47
	Never	189	48.84
Physical activity	Adequate (MET ≥600 per week)	275	71.06
	Inadequate (MET <600 per week)	112	28.94
Sedentary time spent (in minutes)	**Mean (± SD) =** 412.81 **±** 11.88		
	≤4 hours	123	31.78
	>4 hours	264	68.22
Household fuel type for cooking	Clean	46	11.89
	Unclean	341	88.11
Kitchen location	Indoor	189	48.84
	Outdoor	198	51.16
Medical history			
Family history of psychological/mental health issues	No	374	96.64
	Yes	13	3.36
Duration of disease in months (mean±SD)	Diabetes	79.24 (± 3.40)	
	Hypertension	89.48 (± 3.82)	
Regular medicine intake (for diabetes)	No	166	42.89
	Yes	221	57.11
Regular medicine intake (for hypertension)	No	191	49.35
	Yes	196	50.65
Clinical parameters			
Controlled blood pressure	No	344	88.99
	Yes	43	11.11
Controlled blood sugar	No	233	60.21
	Yes	154	39.79
BMI status	Underweight/normal	105	27.13
	Overweight/obese	282	72.87

BMI, body mass index; MET, Metabolic Time Equivalent of Task. MET is defined as the ratio of the energy expended during a specific physical activity to the energy expended at rest.

Regarding behavioural and lifestyle factors, 24.55% of the respondents reported using tobacco in any form (smoke/smokeless). A little over half (51.42%) of the participants consumed less than five groups of food and had inadequate dietary diversity. The majority (71.06%) of the respondents performed adequate physical activity. Mean sedentary time spent per day by the respondents was 412.81 (**±** 11.88) min (table 1).

Only 13 participants (3.36%) reported family history of any psychological or mental health issues. The mean duration of diabetes and hypertension was 79.24 (± 3.40) months and 89.48 (± 3.82) months, respectively. Among the clinical parameters assessed, only 11.11% and 39.79% of participants achieved controlled blood pressure and controlled blood sugar levels, respectively. Regarding BMI, the proportion of overweight and obese participants was 72.87% (table 1).

**Figure 1:**
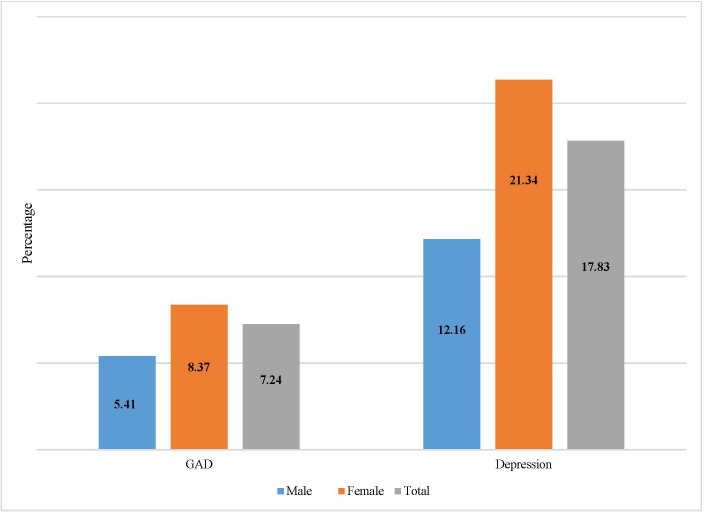
Prevalence of GAD and depression among adults living with diabetes and hypertension comorbidity. GAD, generalised anxiety disorder.

Among 387 participants, 7.24% (95% CI 5.04 to 10.29) had anxiety and 17.83% (95% CI 14.32 to 21.98) had depression (online supplemental table 2). Notably, a higher proportion of female than male had GAD (female: 8.37% vs male: 5.41%) and depression (female: 21.34% vs male: 12.16%) (figure 1). Among the married participants, 15.96% had depression while the participants who did not have any partner had significantly higher prevalence of depression (29.09%, p=0.018) (online supplemental table 2).

### Factors associated with GAD

Table 2 shows that among the participants, age was not significantly associated with GAD. Participants who had complete primary education and incomplete secondary education (grades 5–9) had higher odds of having GAD (adjusted OR (AOR): 3.40, 95% CI 1.26 to 9.19, p=0.016) compared with those who received no education or had incomplete primary education (grades 0–4). People in the highest wealth tertile showed lower odds of having GAD (AOR: 0.12, 95% CI 0.02 to 0.62, p=0.011). Age, physical activity, sedentary time spent per day, and overweight or obesity were not associated with GAD. We also did not find any association of dietary habits like minimum dietary diversity, savoury or sweet intake with GAD.

**Table 2:** Unadjusted and adjusted logistic regression models to find the significant factors associated with GAD and depression among adults living with diabetes and hypertension comorbidity

Characteristics	GAD				Depression			
	OR (95% CI)	P value	AOR	P value	OR (95% CI)	P value	AOR	P value
Age (years)	0.97 (0.94 to 1.01)	0.109	0.99 (0.95 to 1.03)	0.741	0.99 (0.97 to 1.02)	0.598	0.99 (0.96 to 1.02)	0.554
Sex (Ref—male)								
Female	1.60 (0.69 to 3.73)	0.278	2.13 (0.74 to 6.09)	0.157	1.96 (1.09 to 3.51)	0.024	1.20 (0.38 to 3.83)	0.752
Marital status (Ref—currently married)								
Others	0.71 (0.21 to 2.43)	0.584	NA	NA	2.16 (1.13 to 4.14)	0.021	1.80 (0.84 to 3.83)	0.128
Employment (Ref—currently employed)								
Homemaker	1.16 (0.50 to 2.69)	0.730	NA	NA	2.09 (1.09 to 4.00)	0.026	1.48 (0.50 to 4.35)	0.475
Currently unemployed*	0.52 (0.06 to 4.26)	0.538	NA	NA	3.52 (1.29 to 9.59)	0.014	3.26 (1.05 to 10.10)	**0.040**
Retired	0.44 (0.05 to 3.65)	0.450	NA	NA	1.22 (0.37 to 4.01)	0.744	1.34 (0.36 to 4.98)	0.663
Education level (Ref—grades 0–4)								
Grades 5–9†	2.85 (1.14 to 7.14)	0.025	3.40 (1.26 to 9.19)	**0.016**	0.79 (0.42 to 1.50)	0.470	0.97 (0.49 to 1.94)	0.933
Grade ≥10	1.25 (0.44 to 3.53)	0.680	2.40 (0.70 to 8.21)	0.164	0.59 (0.31 to 1.12)	0.106	0.88 (0.39 to 1.96)	0.752
Household wealth status (Ref—lowest)								
Middle	1.21 (0.53 to 2.72)	0.649	1.09 (0.45 to 2.65)	0.853	0.97 (0.54 to 1.77)	0.931	1.02 (0.54 to 1.93)	0.952
Highest*†	0.15 (0.03 to 0.71)	0.016	0.12 (0.02 to 0.62)	**0.011**	0.44 (0.22 to 0.89)	0.022	0.45 (0.21 to 0.98)	**0.044**
Behavioural risk factors								
Tobacco (smokeless/smoking) consumption (Ref—no)								
Yes	1.50 (0.66 to 3.45)	0.335	NA	NA	0.91 (0.49 to 1.69)	0.772	NA	NA
Minimum dietary diversity (Ref—no)								
Yes	1.06 (0.49 to 2.29)	0.876	NA	NA	0.83 (0.50 to 1.41)	0.504	NA	NA
Processed food intake								
Consumption of savoury snacks (Ref—no)								
Yes	0.56 (0.22 to 1.41)	0.217	NA	NA	0.59 (0.32 to 1.08)	0.085	0.72 (0.37 to 1.41)	0.337
Consumption of sweet (Ref—no)								
Yes	2.66 (1.23 to 5.79)	0.013	2.27 (0.96 to 5.39)	0.062	1.00 (0.56 to 1.78)	0.993	1.31 (0.69 to 2.48)	0.402
Consumption of sugar sweetened beverages (Ref—no)								
Yes	0.84 (0.35 to 2.04)	0.699	NA	NA	0.60 (0.32 to 1.13)	0.111	0.75 (0.37 to 1.52)	0.428
Excess salt intake (Ref—never)								
Always/often	1.20 (0.37 to 3.90)	0.757	NA	NA	1.95 (0.92 to 4.11)	0.080	1.95 (0.87 to 4.35)	0.103
Sometimes/rarely	1.33 (0.58 to 3.05)	0.500	NA	NA	1.50 (0.84 to 2.67)	0.168	1.79 (0.96 to 3.33)	0.068
Total physical activity (Ref—adequate)								
Inadequate	0.39 (0.13 to 1.14)	0.086	0.59 (0.18 to 1.90)	0.376	0.84 (0.47 to 1.52)	0.565	NA	NA
Sedentary time spent (Ref—≤4 hours)								
>4 hours†	0.51 (0.23 to 1.11)	0.089	0.58 (0.24 to 1.41)	0.233	0.78 (0.45 to 1.35)	0.382	NA	NA
Fuel type for cooking (Ref—clean)								
Unclean	1.13 (0.33 to 3.92)	0.842	NA	NA	1.24 (0.53 to 2.90)	0.623	NA	NA
Kitchen location (Ref—indoor)								
Outdoor	0.60 (0.27 to 1.31)	0.196	0.43 (0.18 to 1.02)	0.055	0.91 (0.54 to 1.54)	0.729	NA	NA
Duration of disease								
Diabetes	1.00 (0.99 to 1.00)	0.547	NA	NA	1.00 (1.00 to 1.01)	0.203	NA	NA
Hypertension	1.00 (0.99 to 1.00)	0.602	NA	NA	1.00 (1.00 to 1.00)	0.770	NA	NA
Regular medicine intake (for diabetes) (Ref—yes)								
No	1.36 (0.63 to 2.94)	0.432	NA	NA	1.11 (0.65 to 1.87)	0.707	NA	NA
Regular medicine intake (for hypertension) (Ref—yes)								
No	1.03 (0.48 to 2.22)	0.943	NA	NA	0.93 (0.55 to 1.56)	0.779	NA	NA
Clinical parameters								
Controlled blood pressure (Ref—no)								
Yes*	0.96 (0.28 to 3.31)	0.945	NA	NA	2.88 (1.44 to 5.74)	0.003	3.88 (1.81 to 8.35)	0.001
Controlled blood sugar (Ref—no)								
Yes	0.70 (0.31 to 1.59)	0.393	NA	NA	0.90 (0.53 to 1.53)	0.693	NA	NA
BMI status (Ref—underweight/normal)								
Overweight/obese	0.55 (0.25 to 1.21)	0.138	0.56 (0.23 to 1.38)	0.209	0.82 (0.46 to 1.45)	0.496	NA	NA

NA = not applicable; these variables were dropped from the final model because of having p>0.2 in the forward stepwise regression model.

* p value<0.05 for depression.

† p value<0.05 for GAD.

AOR, adjusted OR; BMI, body mass index; GAD, generalised anxiety disorder.

### Factors associated with depression

In our study, we found that unemployment, belonging to the highest wealth tertile and controlled blood pressure were associated with depression. Compared with currently employed participants, unemployed participants had significantly higher odds of having depression (AOR: 3.26, 95% CI 1.05 to 10.10, p=0.040). Participants from the highest wealth tertile had significantly lower odds of depression (AOR: 0.45, 95% CI 0.21 to 0.98, p=0.044). Behavioural/lifestyle factors like tobacco consumption, dietary diversity, processed food (savoury snacks, sweet, sugar sweetened beverages) intake, physical activity, sedentary time spent per day, and overweight or obesity were not associated with depression. The duration of diabetes and hypertension also showed no association with depression. Another finding from the study was that individuals with controlled blood pressure had almost four times higher odds of having depression (AOR: 3.88, 95% CI 1.81 to 8.35, p=0.001) compared with participants without controlled blood pressure. We did not find any statistically significant association of BMI status of individuals with depression.

## Discussion

In this study, we determined the prevalence of GAD and depression among adults living with diabetes and hypertension comorbidity in rural Bangladesh. We found that people living with diabetes and hypertension comorbidity had higher prevalence of GAD and depression compared with the general adult population. We reported a significant association of household wealth status with both GAD and depression. Furthermore, we found education was associated with GAD, and unemployment and controlled blood pressure were associated with depression.

In our study, the prevalence of GAD and depression among adults living with hypertension and diabetes comorbidity is higher than the prevalence of GAD (4.2%) and depressive disorder (6.7%) among the general adult population in Bangladesh.^11^ Previous studies^9 12 13 24^ have also reported higher prevalence of MHDs among people with NCDs. For example, a study by Sekhri and Verma, done in India, reported 49% prevalence of depression among participants with diabetes and/or hypertension.^12^ Another study from India reported 67% prevalence of mild to severe depression among participants with diabetes and hypertension comorbidity.^25^ Although the prevalence varies across studies, likely due to differences in study settings and tools used, the overall findings imply that coexistence of diabetes and hypertension leads to a higher risk of MHDs.

We also found that a higher proportion of women had GAD symptoms and depression compared with men. This gender disparity is consistent with trends observed in other studies, which show that women were more susceptible to MHDs whether in the presence of any comorbidity^15 22 26^ or in the general population.^11^ The heightened vulnerability of women may be attributed to a combination of factors including gender roles, inequalities, caregiving responsibilities, financial dependency and overall societal positionality of women.^27^ While our study relied on a quantitative approach, a qualitative exploration might give a better insight into this scenario.

Household wealth status was significantly associated with both GAD and depression. Participants from the highest wealth tertile exhibited significantly lower odds of GAD as well as depression, reiterating the protecting effect of higher socioeconomic status for mental health disorders.^3 12^ Individuals from the highest wealth tertile are more likely to afford healthcare and regular medications. In contrast, people from lower socioeconomic status struggle more with the out-of-pocket expenses for healthcare, resulting in financial strain and psychological distress.^28^ The financial burden for healthcare is greater on comorbid people due to high medical costs of more than one chronic disease. Lower socioeconomic status may lead to catastrophic health expenditure for this population.^29^ Financial insecurity may also compel individuals to cut back on essential expenses such as nutritious food or medications giving rise to chronic stress, a key risk factor for both GAD and depression.^30^ Accordingly, individuals from higher socioeconomic status may face lower risk of GAD and depression as their better financial stability, compared with people from the lowest wealth tertile, mitigates the stressor resulting from economic hardship. Additionally, other socioeconomic factors, such as education level and employment status, are closely intertwined with wealth status and may also play a critical role in the onset of MHDs in this population.^28^

In our study, we observed participants with 5–9 grades of education had higher odds of GAD. Our study finding contrasts with previous research, which generally suggests that lower educational attainment is a risk factor for anxiety disorders.^20 24^ Education is often linked to greater health awareness, access to healthcare and coping mechanisms, which theoretically should reduce the risk of MHDs. However, our results suggest a nonlinear association between education and GAD, where individuals with moderate levels of education (grades 5–9) were more vulnerable compared with both lower (grades 0–4) and higher (grade ≥10) educated groups. One possible explanation is that individuals with a moderate level of education but without secondary completion may face higher psychological stress due to limited job opportunities, financial instability and societal expectations. They may have higher career expectation than those with little or no education, but lack the qualification needed to have opportunities in the formal and informal job market. As a result, they may experience greater frustration due to underemployment or lack of career advancement opportunities.^31^ Some authors have highlighted a bidirectional relationship between mental health and education, that is, higher educational attainment can both protect against and contribute to anxiety, depending on socioeconomic context and job market conditions.^31^

We also found that unemployment and controlled blood pressure were two factors associated with depression. In our study, participants who were unemployed demonstrated higher odds of depression, consistent with the prior evidence that links joblessness with poor mental health outcome.^15 22^ Unemployment, low socioeconomic condition and mental health can be interlinked. A study by Emre *et al* reported an inverse association only with the prevalence of depression and income but not with anxiety.^27^ Loss of social status, uncertainty and financial insecurity are associated with unemployment, contributing to the development of depression.^32 33^ An unexpected finding from our study was that participants with controlled blood pressure had higher odds of depression. This finding might be due to the effect of antihypertensive drugs.^34^ A systematic review and meta-analysis reported that antihypertensive drugs like calcium channel blockers and beta-blockers might act as potential risk factors for depression.^35^ However, existing literature regarding this association is inconclusive as some other studies suggested neutral or even protective effect of antihypertensive drugs on mental health outcomes.^36 37^ Therefore, our finding warrants further research among this population, to explore if any other biological or physiological factors or medication-related mechanisms are attributing to this association.

We further explored behavioural factors such as dietary habits, physical activity and tobacco consumption for association with GAD and depression. Contrary to the existing literature, these factors did not show any significant associations with GAD or depression in our study. Usually, hypertension, diabetes and even depression share common behavioural risk factors.^38^ For example, authors from several studies reported that insufficient physical activity and inadequate dietary diversity are linked with both anxiety and depressive symptoms in diabetic patients.^22 39^ Another study among diabetes and hypertension comorbid population showed significant association of physical activity and smoking with depression.^3^ However, a systematic review of longitudinal studies showed inconclusive results in prospective association between smoking and anxiety or depression.^40^ Among the 148 studies included in this review, some studies showed positive association between smoking and MHDs, some found a reverse association between MHDs and smoking and some studies reported no association.^40^ Though some of these study findings are in alignment with our findings of no association of smoking with anxiety or depression, further research is needed to develop a better understanding of this complex association among individuals living with hypertension and diabetes comorbidity.

Additionally, the duration of disease, controlled blood sugar level and BMI status were not found to be associated with either GAD or depression. Consistent with our study result, another study also failed to establish any association between duration of diabetes and anxiety or depression.^39^ However, several studies have identified a correlation between the duration of diabetes and the prevalence of depression and anxiety.^1220 41^,^43^ This discrepancy may be attributable to the unique sociocultural context of Bangladesh, where the influence of chronic illness on mental health may manifest differently. Differences in illness perception, beliefs about disease management, or adaptive coping mechanisms developed over long-term illness may influence the chance of having GAD or depression among this population.^44^

Despite the mixed findings, this study has its own strength as it gives novel insight into mental health status of people living with diabetes and hypertension comorbidity. This is the first study, to our knowledge, to assess GAD and depression and explore associated factors among this unique comorbid population in Bangladesh, providing evidence for integrating mental health services into NCD management programmes at the primary healthcare level. However, the study has some limitations. First, as the study design is cross-sectional, we could not establish any causal relationship between the factors and GAD and depression. Second, there is a possibility of information bias due to the self-reported nature of responses. Third, both GAD-7 and PHQ-9 tools are used for screening purposes but do not confirm the clinical diagnosis. Fourth, some confounders such as family relationships, social support and coping mechanisms could have been explored in depth with a qualitative approach in a mixed-method study design to better understand their context and untangle the complex relation of socioeconomic and other factors contributing to mental health.

## Conclusion

We conclude that the prevalence of GAD and depression among this population with diabetes and hypertension comorbidity was higher compared with the general population in Bangladesh. As the Bangladesh government has already included mental health disorders under the non-communicable disease control programme, this study provides evidence for the importance of integrating mental health interventions into the NCD care management at primary healthcare level. Socioeconomic characteristics like education level and household wealth status were significant factors for GAD while unemployment and household wealth status emerged as factors significantly associated with depression. The higher odds of depression among the study participants with controlled blood pressure warrant further research. We also observed that behavioural factors such as tobacco consumption, inadequate physical activity and unhealthy dietary habits were not associated with either GAD or depression. Further longitudinal studies are warranted to better understand the causal association between these factors and GAD and depression. However, findings from our study will be crucial to develop targeted interventions for the identification and management of MHDs among people with hypertension and diabetes comorbidity.

## Supplementary material

10.1136/bmjopen-2025-102000 online supplemental file 1

## Data Availability

Data are available upon reasonable request.
