# Supplementary material for: Factors associated with generalised anxiety disorder and depression among adults living with diabetes and hypertension comorbidity in rural Bangladesh: findings from a cross-sectional study
Source: BMJ Open. 2025 Sep 5;15(9):e102000. doi: 10.1136/bmjopen-2025-102000 (PMC12414191; doi:10.1136/bmjopen-2025-102000)
Supplement: online supplemental file 1 [file bmjopen-15-9-s001.docx]

**Supplementary materials**

**Supplementary table 1: Categories/measurements of explanatory variables of this study**

| **Domain** | **Variables** | **Categories/measurements** |
| --- | --- | --- |
| Socio-demographic information | Age | 18 – 39 years, 40 – 49 years, 50-59 years,  60 years and above |
|  | Sex | Male, Female |
|  | Marital status | Currently married  Others: included never married, divorced, widowed, separated, and cohabitating |
|  | Employment | Currently employed, homemaker, currently unemployed, retired |
|  | Religion | Islam  Others: included Hindu, Christian, Buddhist, and other religions |
|  | Education level | Grades 0-4, grades 5-9, grades 10 or higher |
|  | Household wealth status | Lowest, middle, highest |
| Behavioural risk factors | Tobacco (smoking/smokeless) consumption | Any smokeless or smoking tobacco consumption in the last one month (yes, no) |
|  | Minimum dietary diversity | Consumption of food from at least 5 or more out of 10 specific food groups in previous 24 hours was considered adequate dietary diversity (22). (inadequate, adequate) |
|  | Consumption of savory snacks | Yes, no |
|  | Consumption of sweet | Yes, no |
|  | Consumption of sugar sweetened beverages | Yes, no |
|  | Excess salt intake | Always/often, sometimes to rarely, never |
|  | Physical activity | An equivalent combination of moderate and vigorous intensity physical activity achieving at least 600 MET was considered adequate (23). (Inadequate, adequate) |
|  | Sedentary time spent (in minutes) | time spent sitting or lying with low energy expenditure, while awake, in the context of occupational, educational, home and community settings, and transportation (24). |
|  | Household fuel type for cooking | Clean: Electricity (including solar panels), piped natural gas, liquefied petroleum gas, biogas;  Unclean: Alcohol/ethanol, gasoline/diesel, kerosene/paraffin, coal/ignite, charcoal, processed biomass pellets, agricultural crops/straw/shrubs/corn cobs/wood chips/sawdust/wood/animal waste/dung, garbage/plastic. |
|  | Kitchen location | Outdoor, indoor |
| Medical history and self-care practices | Family history of psychological/mental health issues | Yes, no |
|  | Duration of disease (in months) | Diabetes: Duration since the participant was diagnosed with diabetes; hypertension: duration since the participant was diagnosed with hypertension. |
|  | Regular medicine intake (for diabetes) | Yes, no |
|  | Regular medicine intake (for hypertension) | Yes, no |
| Clinical parameters | Controlled blood pressure | Yes: BP ≤130/80mmHg (25); no |
|  | Controlled blood sugar | Yes: Random blood sugar level ≤11.1mmol/l (25); no |
|  | Body mass index (BMI) status | Using Asian cut-off as followings: underweight/normal (BMI <18.5kg/m^2^/18.5-22.9 kg/m^2^), overweight or obese (BMI≥23.0 kg/m^2^) (26). |

**Supplementary table 2: Prevalence of GAD and depression by background characteristics (n=387)**

| **Characteristics** | **GAD status** | | **Depression status** | |
| --- | --- | --- | --- | --- |
|  | **Yes (%)** | **p-value** | **Yes (%)** | **p-value** |
| **Overall** | 7.24  (95% CI: 5.04 to 10.29) |  | 17.83  (95% CI:  14.32 to 21.98) |  |
| **Age (years) (Mean±SD)** | 53.75±13.71 | 0.107 | 56.36±12.33 | 0.599 |
| **Sex** |  |  |  |  |
| Male | 5.41 | 0.274 | 12.16 | 0.022 |
| Female | 8.37 |  | 21.34 |  |
| **Marital status** |  |  |  |  |
| Currently Married | 7.53 | 0.582 | 15.96 | 0.018 |
| Others | 5.45 |  | 29.09 |  |
| **Employment** |  |  |  |  |
| Currently Employed | 7.20 | 0.695 | 11.20 | 0.038 |
| Homemaker | 8.25 |  | 20.87 |  |
| Currently unemployed | 3.85 |  | 30.77 |  |
| Retired | 3.33 |  | 13.33 |  |
| **Education level** |  |  |  |  |
| Grade 0-4 | 4.82 | 0.045 | 9.23 | 0.262 |
| Grade 5-9 | 12.62 |  | 10.94 |  |
| Grade ≥10 | 5.93 |  | 1.55 |  |
| **Wealth index** |  |  |  |  |
| Lowest | 9.23 | 0.008 | 21.54 | 0.040 |
| Middle | 10.94 |  | 21.09 |  |
| Highest | 1.55 |  | 10.85 |  |
| **Behavioural risk factors** |  |  |  |  |
| **Tobacco (smokeless/smoking) consumption** | | | | |
| No | 6.51 | 0.332 | 18.15 | 0.772 |
| Yes | 9.47 |  | 16.84 |  |
| **Minimum dietary diversity** |  |  |  |  |
| No | 7.04 | 0.024 | 18.75 | 0.887 |
| Yes | 7.45 |  | 17.75 |  |
| **Savory snacks intake** | | | | |
| No | 8.37 | 0.211 | 20.15 | 0.104 |
| Yes | 4.84 |  | 12.90 |  |
| **Sweet intake** | | | | |
| No | 5.09 | 0.011 | 17.82 | 0.993 |
| Yes | 12.50 |  | 17.86 |  |
| **Drinks** | | | | |
| No | 7.55 | 0.699 | 19.78 | 0.109 |
| Yes | 6.42 |  | 12.84 |  |
| **Excess salt intake** | | | | |
| Always/often | 7.55 | 0.793 | 24.53 | 0.156 |
| Sometimes to rare | 8.28 |  | 20.00 |  |
| Never | 6.35 |  | 14.29 |  |
| **Total physical activity** |  |  |  |  |
| Adequate | 8.73 | 0.076 | 18.55 | 0.564 |
| Inadequate | 3.57 |  | 16.07 |  |
| **Sedentary time spent** |  |  |  |  |
| ≤4hrs | 10.57 | 0.084 | 20.33 | 0.381 |
| >4hrs | 5.68 |  | 16.67 |  |
| **Fuel type** |  |  |  |  |
| Clean | 6.52 | 0.842 | 15.22 | 0.622 |
| Unclean | 7.33 |  | 18.18 |  |
| **Kitchen location** |  |  |  |  |
| Indoor | 8.99 | 0.192 | 18.52 | 0.729 |
| Outdoor | 5.56 |  | 17.17 |  |
| **Family history of psychological/mental health issues** | | | | |
| Yes | 7.69 | 0.948 | 23.08 | 0.615 |
| No | 7.22 |  | 17.65 |  |
