# Supplementary material for: Factors associated with generalised anxiety disorder and depression among adults living with diabetes and hypertension comorbidity in rural Bangladesh: findings from a cross-sectional study
Source: BMJ Open. 2025 Sep 5;15(9):e102000. doi: 10.1136/bmjopen-2025-102000 (PMC12414191; doi:10.1136/bmjopen-2025-102000)
Supplement: Supplementary file 2 [file bmjopen-2025-102000.reviewer_comments.pdf]

PEER REVIEW HISTORY

BMJ Open publishes all reviews undertaken for accepted manuscripts. Reviewers are asked to complete a checklist review form and are provided with free text boxes to elaborate on their assessment. These free text comments are reproduced below.

ARTICLE DETAILS

Title (Provisional)

Factors Associated with Generalized Anxiety Disorder and Depression among Adults living with Diabetes and Hypertension Comorbidity in Rural Bangladesh: Findings from a Cross-Sectional Study

Authors

Chakravartty, Meghna; Shuvo, Md. Mashuk Shahriar; Kumari, Sita; Jhilik, Tanni Chakma; Sarker, Tanmoy; Akter, Fahmida; Hossain, Md Mokbul; Ahsan, Ali; Latif, Mahbub; Mridha, Malay Kanti

---

VERSION 1 - REVIEW

**Reviewer**	**1**
**Name**	**Ariaratnam, Suthahar**
**Affiliation**	**Universiti Teknologi MARA, Faculty of Medicine**
**Date**	**07-Apr-2025**
**COI**	**None**

---

1. Authors need to write in formal English:

Some examples:

a. Expand on acronym “NCD” (under introduction: line 5)

Expand on acronym …(LMICs)…. (under introduction: line 12)

b. Many sentences used the word “and” at least twice. Indeed, some had “and” on 3 occasions in one sentence. Kindly, rewrite into 2-3 sentences to improve clarity or add while or whilst where applicable.

c. Ethical approval for the study was obtained from the 6 Informed consent was obtained… (line 5 pg 10). This sentence is unclear. Kindly, rewrite it to improve clarity. Also, try to avoid 2 words of “was” in one sentence.

d. Use past tense in most of the manuscript if applicable.

e. “The prevalence of GAD (4.2%) and depressive disorder (6.7%) among general adult population in

Bangladesh (11), is lower than the prevalence among adults living with hypertension and diabetes

comorbidity, found in our study". Better to rewrite as "in our study, the prevalence of.....

f. Under discussion, when reporting on gender differences, use new paragraph.

2. Authors need to justify reason/s of exploring GAD as a specific diagnosis entity while depression was nonspecific. Why did not explore major depressive disorders or persistent depressive disorders etc as a more specific diagnostic entities to harmonize with GAD.

3. Brief description of both General Anxiety Disorder-7 (GAD-7) and Patient Health Questionnaire-9 (PHQ-9). Additionally, any validation of these questionnaires in the local context.

4. …data analysis using software STATA 17.0…..Any reference for this? Please cite them in text as well.

5. Under Ethics section, any approval from the regional ethics committee would be helpful. If none, kindly state reason/s

6. Discussion occasionally overlaps and is irrelevant. Some of the data on para 1 can be omitted including "We observed that the common behavioral risk factors and clinical variables were not associated GAD or depression" can be misleading and better omitted.

7. Under discussion (page 19 para 2), appropriate to mention only the present study findings (which are significant) and why the present study differed from previous studies.

**Reviewer**	**2**
**Name**	**SALINERO-FORT, MIGUEL**
**Affiliation**	**Foundation for Biomedical Research and Innovation in Primary Care of the Community of Madrid**
**Date**	**06-Jun-2025**
**COI**	**None**

The manuscript has a clear and well-defined objective, which is timely considering the limited information available on the prevalence of depression and anxiety among individuals with diabetes and hypertension in South Asian countries. Specifically, the Bangladeshi population is generally younger than those in Europe, America, and other Asian nations such as Japan. Therefore, it is crucial to determine the proportion of patients with diabetes and hypertension who also experience depression and anxiety. These chronic conditions significantly increase the risk of cardiovascular complications and renal damage. The

coexistence of these diseases with anxiety and depression can lead to poorer health outcomes and heightened mortality rates.

Major considerations:

The methodology employed in this study is appropriate, but clarification is needed in several areas. Firstly, it is unclear how participants with diabetes and hypertension were identified. Were they identified through interviews, diagnosed based on medical history, or through measurement of specific parameters? Secondly, have the questionnaires used for screening depression and anxiety been previously validated in Bangladesh? It is important to note that the cultural patterns of the South Asian population differ significantly from those of Anglo-Saxon countries. Consequently, cross-cultural adaptation is necessary—this process involves modifying the questionnaire to ensure it is suitable for the cultural context of the South Asian population. Additionally, determining appropriate cut-off points for this demographic is essential. Moreover, since English is not the first language in the country, it should be clarified whether the questionnaires were administered in English. For these reasons, it is crucial to confirm that the questionnaires are sufficiently validated, highlighting the importance of linguistic validation in research. Furthermore, while the report discusses depression and anxiety, it is important to note that these questionnaires should not be used for diagnostic purposes, as they can result in false positives and false negatives. They possess specific sensitivity and specificity in relation to a reference standard, such as the DSM-5. However, from an epidemiological perspective, a positive result on a questionnaire is typically interpreted as indicative of the presence of the condition.

Minor considerations:

The 95% confidence intervals for depression and anxiety have been miscalculated. Additionally, some results are duplicated in the text, and there are grammatical errors that need to be corrected. Table 1 includes information that is not particularly relevant and could be moved to a supplementary table. Furthermore, Table 2 presents results that are excessively repeated in both the text and the table. I recommend including only the most relevant information in the main text. There is a comment in the Results section about the surprising positive association between blood pressure control and depression, which is discussed in detail in the Discussion section. Therefore, references from the Discussion section should be avoided in the Results section. The ethics committee that approved the study and the reference number are not specified.

VERSION 1 - AUTHOR RESPONSE

Reviewer 1

Reviewer: 1

Dr. Suthahar Ariaratnam, Universiti Teknologi MARA, Universiti Teknologi MARA
Comments to the Author:

Comment 1. Authors need to write in formal English:
Some examples:
a. Expand on acronym "NCD" (under introduction: line 5)
Expand on acronym …(LMICs)…. (under introduction: line 12)
b. Many sentences used the word "and" at least twice. Indeed, some had "and" on 3 occasions in one sentence. Kindly, rewrite into 2-3 sentences to improve clarity or add while or whilst where applicable.
c. Ethical approval for the study was obtained from the 6 Informed consent was obtained… (line 5 pg 10). This sentence is unclear. Kindly, rewrite it to improve clarity. Also, try to avoid 2 words of "was" in one sentence.
d. Use past tense in most of the manuscript if applicable.
e. "The prevalence of GAD (4.2%) and depressive disorder (6.7%) among general adult population in
Bangladesh (11), is lower than the prevalence among adults living with hypertension and diabetes
comorbidity, found in our study". Better to rewrite as "in our study, the prevalence of…..
f. Under discussion, when reporting on gender differences, use new paragraph.

Response: Thank you for the valuable suggestions. We have carefully revised the manuscript throughout to improve grammar and use of formal English.
a. Acronyms such as "NCD" (non-communicable disease) and "LMICs" (low- and middle-income countries) have now been expanded at first mention in the introduction. (Please see page 4)

b. We have revised the manuscript and long sentences with multiple use of "and" have been split into shorter, clearer statements. (Please see page 4).

Please see below the made changes in page 4 in the main manuscript (marked copy):

'This coexistence of diabetes and hypertension can lead to serious health outcomes like heart attacks and stroke, kidney function impairment etc. and adequate management and control over both conditions is necessary for prevention of those health complications (8).' – **This sentence was split into two sentences and rewritten as following:**

'This coexistence of diabetes and hypertension can lead to serious health outcomes like heart attacks and stroke, kidney function impairment etc. Adequate management and control over both conditions is necessary for prevention of those health complications (8).' (Please see page 4, under Introduction section)

c. The unclear sentence on page 10 has been corrected and rewritten as: *"Ethical approval for the study was obtained from the Institutional Review Board of BRAC JPGSPH (Reference no: MPH-2024-007). Informed consent was obtained from all participants, assuring their*

voluntary participation and autonomy to withdraw at any time." (Please see page 10, under sub-heading Ethics, Section: Methods)

d. The manuscript has been further reviewed to ensure consistent use of past tense, where appropriate.

e. The sentence comparing prevalence has been revised as:
"In our study, the prevalence among adults living with hypertension and diabetes comorbidity is higher than the prevalence of GAD (4.2%) and depressive disorder (6.7%) among general adult population in Bangladesh (11)." (Please see page 18, section: Discussion)

f. A new paragraph has been incorporated in the discussion section in which gender-based differences are discussed as suggested. (Please see page 16, 2nd paragraph, section: Discussion)

Comment 2. Authors need to justify reason/s of exploring GAD as a specific diagnosis entity while depression was nonspecific. Why did not explore major depressive disorders or persistent depressive disorders etc as a more specific diagnostic entities to harmonize with GAD.
Response: Thank you for this important comment. We have now included a justification in the "Outcome Measures" section of the Methods. GAD was assessed as a distinct anxiety disorder using the GAD-7 scale because it is a well-established, specific screening tool. In contrast, depression was measured using PHQ-9 scale to capture a broader spectrum of depressive symptoms without differentiating into clinical subtypes like major depressive disorder or persistent depressive disorder. Moreover, GAD-7 and PHQ-9 tools are widely used and validated to capture data in community settings globally. This approach is common in epidemiological studies, particularly in LMIC settings, and aligns with our aim to identify individuals with generalized anxiety or depressive symptoms who may require support, rather than make clinical diagnoses. (Please see page 6, under Outcome measures subheading in Methods section)

Comment 3. Brief description of both General Anxiety Disorder-7 (GAD-7) and Patient Health Questionnaire-9 (PHQ-9). Additionally, any validation of these questionnaires in the local context.
Response: A description of both GAD-7 and PHQ-9 tools have been added in the Methods section. We also cited relevant validation studies from Bangladesh for the GAD-7 and PHQ-9 tools used. We have also added a description of the pretesting process of the questionnaire. The questionnaire was pre-tested in Birganj (a nearby sub-district in Dinajpur). (Please see page 6, sub-section: Outcome measures, section: Methods)

Comment 4. …data analysis using software STATA 17.0…..Any reference for this? Please cite them in text as well.
Response: Thank you. We used STATA 17.0 (StataCorp, Texas 77845 USA) and included

this reference in Methods section. (Please see page 10, Statistical analysis sub-section, under Methods section)

Comment 5. Under Ethics section, any approval from the regional ethics committee would be helpful. If none, kindly state reason/s
Response: Thank you for the observation. We have revised the ethics statement including the full name of the ethics committee and the approval reference number. Our study got the ethical approval from the Institutional Review Board of BRAC JPGSPH (Reference no: MPH-2024-007). (Please see page 10. Sub-section: Ethics, Section: Methods)

Comment 6. Discussion occasionally overlaps and is irrelevant. Some of the data on para 1 can be omitted including "We observed that the common behavioral risk factors and clinical variables were not associated GAD or depression" can be misleading and better omitted.
Response: Thank you for this valuable comment. In the first paragraph of the Discussion section, we aimed to summarize the key findings of the study. As per your suggestion, we have omitted the sentence "We observed that the common behavioral risk factors and clinical variables were not associated with GAD or depression," as it could be misinterpreted or seen as misleading. (Please see page 18, Discussion section)

Comment 7. Under discussion (page 19 para 2), appropriate to mention only the present study findings (which are significant) and why the present study differed from previous studies.
Response: We revised the Discussion section as per your suggestion. (Please see page 20-21, Discussion section)

Reviewer 2

Reviewer: 2
Dr. MIGUEL SALINERO-FORT, Foundation for Biomedical Research and Innovation in Primary Care of the Community of Madrid, IdiPAZ

General comment:
The manuscript has a clear and well-defined objective, which is timely considering the limited information available on the prevalence of depression and anxiety among individuals with diabetes and hypertension in South Asian countries. Specifically, the Bangladeshi population is generally younger than those in Europe, America, and other Asian nations such as Japan. Therefore, it is crucial to determine the proportion of patients with diabetes and hypertension who also experience depression and anxiety. These chronic conditions significantly increase the risk of cardiovascular complications and renal damage. The coexistence of these diseases with anxiety and depression can lead to poorer health outcomes

and heightened mortality rates.
Response: Thank you very much for your comment.

Major considerations:
Comment: The methodology employed in this study is appropriate, but clarification is needed in several areas. **Firstly,** it is unclear how participants with diabetes and hypertension were identified. Were they identified through interviews, diagnosed based on medical history, or through measurement of specific parameters?
Response: Thank you for the observation. We have added details of how sampling frame were created and the participants with diabetes and hypertension were identified in the "Sampling" sub-section under Methods section.

Our study site was Chirirbandar, Dinajpur, Bangladesh. It is a NCD surveillance site under the project titled "Understanding the Patterns and Determinants of Health in South Asian People: South Asia Biobank" of BRAC JPGSPH. In the South Asia Biobank project, individuals with diabetes and hypertension were identified using clinical measurements (blood pressure and fasting blood glucose) following WHO diagnostic criteria. We created our sampling frame from the existing list of individuals identified with diabetes and hypertension in the NCD surveillance system. The sampling frame comprised 481 people with diabetes and hypertension comorbidity. (Please see page 5-6, under Sampling, subheading in Methods section)

Comment: Secondly, have the questionnaires used for screening depression and anxiety been previously validated in Bangladesh? It is important to note that the cultural patterns of the South Asian population differ significantly from those of Anglo-Saxon countries. Consequently, cross-cultural adaptation is necessary—this process involves modifying the questionnaire to ensure it is suitable for the cultural context of the South Asian population. **Additionally,** determining appropriate cut-off points for this demographic is essential. **Moreover,** since English is not the first language in the country, it should be clarified whether the questionnaires were administered in English. For these reasons, it is crucial to confirm that the questionnaires are sufficiently validated, highlighting the importance of linguistic validation in research. **Furthermore,** while the report discusses depression and anxiety, it is important to note that these questionnaires should not be used for diagnostic purposes, as they can result in false positives and false negatives. They possess specific sensitivity and specificity in relation to a reference standard, such as the DSM-5. However, from an epidemiological perspective, a positive result on a questionnaire is typically interpreted as indicative of the presence of the condition.

Response: Thank you for your valuable comment. A short description of tools used (GAD-7 and PHQ-9) have been included in methods section. The description encompasses the tools used (GAD-7 and PHQ-9), scoring systems, and the cut-off points for defining generalized anxiety disorder (GAD) and depression. We have further added a description of the pretesting process and cited relevant validation studies from Bangladesh for the GAD-7 and PHQ-9 tools used. (Please see page 6, sub-section: Outcome measures, section: Methods).

GAD was assessed as a distinct anxiety disorder using the GAD-7 because it is a well-established, specific screening tool. In contrast, depression was measured using PHQ-9 to capture a broader spectrum of depressive symptoms without differentiating into clinical subtypes like major depressive disorder or persistent depressive disorder. This approach is common in epidemiological studies, particularly in LMIC settings, and aligns with our aim to identify individuals with generalized anxiety or depressive symptoms who may require support, rather than make clinical diagnoses. (Please see page 6, sub-section: Outcome measures, section: Methods).

Minor considerations:
Comment: The 95% confidence intervals for depression and anxiety have been miscalculated.
Response: Thank you for the observation. We have corrected the 95% confidence intervals for depression and anxiety as following:

The prevalence of GAD was 7.24% (95% CI: 5.04 to 10.29). (Please see page 2, Abstract)

The prevalence of depression was 17.83% (95% CI: 14.32 to 21.98). (Please see page 2, Abstract)

Among 387 participants, 7.24% (95% CI: 5.04 to 10.29) had anxiety and 17.83% (95% CI: 14.32 to 21.98) had depression (Supplementary table 2). (Please see page 14, section: Results, after Table 1)

Comment: Additionally, some results are duplicated in the text, and there are grammatical errors that need to be corrected. Table 1 includes information that is not particularly relevant and could be moved to a supplementary table.

Response: Thank you for the feedback. We have carefully revised the manuscript correcting the grammatical errors. We also have reviewed and revised the Results section to avoid repetition of information already presented in tables. Only the most relevant findings are now described in the text to maintain clarity and conciseness. (Please see page 11, Result section)

We agree that not all variables presented in Table 1 were essential to the main narrative. As recommended, we have moved Table 1 to the supplementary materials, labelled as Supplementary Table 1. (Please see Supplementary table 1, supplementary materials)

Comment: Furthermore, Table 2 presents results that are excessively repeated in both the text and the table. I recommend including only the most relevant information in the main text.

Response: Thank you for the suggestion. We have carefully reviewed the Results section and Table 2 to eliminate redundancy. Only the most statistically significant and relevant findings from Table 2 are now highlighted in the main text. Descriptive or less relevant details have

been retained in the table but omitted from the narrative to improve clarity and avoid repetition. (Please see page 11-15, Results section).

Moreover, as previously labelled Table 1 has been moved to supplementary material, Table 2 is labelled as Table 1 in the updated revised version. (Please see page 11-15, Results section)

Comment: There is a comment in the Results section about the surprising positive association between blood pressure control and depression, which is discussed in detail in the Discussion section. Therefore, references from the Discussion section should be avoided in the Results section.

Response: Thank you for the observation. We have revised the result section accordingly. (Please see page 15, under Discussion section)

Comment: The ethics committee that approved the study and the reference number are not specified.
Response: Thank you for the observation. We have revised the ethics statement including the full name of the ethics committee and the approval reference number. Our study got the ethical approval from the Institutional Review Board of BRAC JPGSPH (Reference no: MPH-2024-007). (Please see page 10. Sub-section: Ethics, Section: Methods).

Reviewer: 1
If you have selected 'Yes' above, please provide details of any competing interests.: Not applicable

Reviewer: 2
If you have selected 'Yes' above, please provide details of any competing interests.: Not applicable

---

VERSION 2 - REVIEW

**Reviewer**	**2**
**Name**	**SALINERO-FORT, MIGUEL**
**Affiliation**	**Foundation for Biomedical Research and Innovation in Primary Care of the Community of Madrid**
**Date**	**17-Aug-2025**
**COI**	

All the comments raised have been satisfactorily addressed, and the suggested revisions have been incorporated into the manuscript. Consequently, the text now meets the standards for publication, subject to the editor's final judgment. I wish the authors the best of luck.
